# Greater than the parts: a review of the information decomposition approach to causal emergence

**DOI:** 10.1098/rsta.2021.0246

**Published:** 2022-07-11

**Authors:** Pedro A. M. Mediano, Fernando E. Rosas, Andrea I. Luppi, Henrik J. Jensen, Anil K. Seth, Adam B. Barrett, Robin L. Carhart-Harris, Daniel Bor

**Affiliations:** ^1^ Department of Psychology, University of Cambridge, Cambridge, UK; ^2^ University Division of Anaesthesia, University of Cambridge, Cambridge, UK; ^3^ Department of Clinical Neurosciences, University of Cambridge, Cambridge, UK; ^4^ Leverhulme Centre for the Future of Intelligence, University of Cambridge, Cambridge, UK; ^5^ Department of Psychology, Queen Mary University of London, London, UK; ^6^ Centre for Psychedelic Research, Imperial College London, London, UK; ^7^ Data Science Institute, Imperial College London, London, UK; ^8^ Centre for Complexity Science, Imperial College London, London, UK; ^9^ Department of Mathematics, Imperial College London, London, UK; ^10^ The Alan Turing Institute, London, UK; ^11^ Institute of Innovative Research, Tokyo Institute of Technology Tokyo, Japan; ^12^ Sackler Centre for Consciousness Science, University of Sussex, Brighton, UK; ^13^ The Data Intensive Science Centre, Department of Informatics, University of Sussex, Brighton, UK; ^14^ CIFAR Program on Brain, Mind, and Consciousness, Toronto, Canada; ^15^ Psychedelics Division, Neuroscape, Department of Neurology, University of California, San Francisco, CA, USA

**Keywords:** synergy, emergence, information decomposition

## Abstract

Emergence is a profound subject that straddles many scientific disciplines, including the formation of galaxies and how consciousness arises from the collective activity of neurons. Despite the broad interest that exists on this concept, the study of emergence has suffered from a lack of formalisms that could be used to guide discussions and advance theories. Here, we summarize, elaborate on, and extend a recent formal theory of causal emergence based on information decomposition, which is quantifiable and amenable to empirical testing. This theory relates emergence with information about a system’s temporal evolution that cannot be obtained from the parts of the system separately. This article provides an accessible but rigorous introduction to the framework, discussing the merits of the approach in various scenarios of interest. We also discuss several interpretation issues and potential misunderstandings, while highlighting the distinctive benefits of this formalism.

This article is part of the theme issue ‘Emergent phenomena in complex physical and socio-technical systems: from cells to societies’.

## 1. Introduction

Emergence is a key concept in several challenging open questions in science and philosophy, and a subject of long-standing debate. A distinctively controversial topic, research on emergence has been characterized by differing assumptions and positions—explicit and implicit—about its nature and role within science. At one extreme of the spectrum, *reductionism* claims that all that is ‘real’ can always be explained based on sufficient knowledge of a system’s smallest constituents, and that coarse-grained explanations are mere byproducts of our limited knowledge and/or computational ability. At the other extreme, strong forms of *emergentism* argue for a radical independence between layers of reality, such that some high-level phenomena are in principle irreducible to their low-level constituents.

Modern scientific practice is dominated by reductionist assumptions, at least in its overall theoretical and philosophical commitments. At the same time, the hierarchical organization and in-practice relative independence of the domains of different scientific disciplines (e.g. physics, biology) suggests that some form of emergentism remains in play. There is, therefore, a need to formulate principled, rigorous and consistent formalisms of emergence, a need that is especially pressing for those topics where strong emergentism retains intuitive appeal—such as the relationship between consciousness and the brain.

Riding on a wave of renewed philosophical investigations [1,2], recent work is opening a new space of discussion about emergence that is firmly within the realm of empirical scientific investigation [3–9]. This work is developing formal principles and analytical models, which promise to facilitate discussions among the community of interested researchers. Moreover, having a formal theory of emergence will allow scientists to formulate rigorous, falsifiable conjectures about emergence in different scenarios and test them on data.

This article presents an overview of a recently proposed formal theory of causal emergence [7] based on the framework of partial information decomposition (PID) [10]. By contrast with other proposals, this approach is primarily *mereological*: emergence is considered to be a property of part-whole relationships within a system, which depends on the relationship between the dynamics of parts of the system and macroscopic features of interest. In what follows, we outline the necessary mathematical background, present the core principles of the theory, and review some of its key properties and applications.

## 2. Technical preliminaries

### (a) An information-centric perspective on complex systems

Information theory is deeply rooted in probability theory, to the extent that the axiomatic bases of both are formally equivalent [11]. Both approaches, in turn, are illuminated by the seminal work of E. T. Jaynes on the foundations of thermodynamics [12], which proposes that probability theory can be understood as an extension of Aristotelian logic that applies to scenarios of partial or incomplete knowledge. In this context, probability distributions are to be understood as epistemic statements used to represent states of limited knowledge, and Shannon’s entropy corresponds to a fundamental measure of uncertainty.

This perspective leads to principled and broadly applicable interpretations of information-theoretic quantities. In fact, while information theory was created to solve engineering problems in data transmission [13], modern approaches cast information quantities as measures of belief-updating in statistical inference [14–16]. In this view, measuring the mutual information between parts of a complex system does not require assuming one is ‘sending bits’ to the other over some channel—instead, mutual information can be seen as the strength of the evidence supporting a statistical model in which the two parts are coupled (although see [17] for an alternative discussion). Furthermore, information-theoretic tools are widely applicable in practice, spanning categorical, discrete and continuous, as well as linear and nonlinear scenarios. A variety of estimators and open-source software is available, whose diversity in terms of assumptions and requirements allows reliable calculations on a broad range of practical scenarios [18–20].

Together, these properties place information theory as a particularly well-suited framework to study interdependencies in complex systems, establishing information as a ‘common currency’ of interdependence that allows one to assess and compare diverse systems in a principled and substrate-independent manner [21–23].

### (b) The fine art of information decomposition

Shannon’s information is particularly useful for the study of complex systems due to its decomposability. For example, the information about a variable Y provided by two predictors $X_{1}$ and $X_{2}$, denoted by $I(X_{1},X_{2};Y)$, can be decomposed via the *information chain-rule* [24] as

2.1 $$I(X_{1},X_{2};Y)=I(X_{1};Y)+I(X_{2};Y|X_{1}),$$

where $I(X_{1};Y)$ corresponds to the information provided by $X_{1}$, and $I(X_{2};Y|X_{1})$ refers to the information provided by $X_{2}$ when $X_{1}$ is already known. Taking this idea one step further, the PID framework [10] proposes to decompose each of these terms into *information atoms* as follows:

2.2 $$\begin{array}{rl} & I(X_{1};Y)=\text{Red}(X_{1},X_{2};Y)+\text{Un}(X_{1};Y|X_{2})\\ \;\mathrm{and}\; & I(X_{2};Y|X_{1})=\text{Un}(X_{2};Y|X_{1})+\text{Syn}(X_{1},X_{2};Y), \end{array}\}$$

where $\text{Red}(X_{1},X_{2};Y)$ represents the *redundant* information about Y that is contained in both $X_{1}$ and $X_{2}$, $\text{Un}(X_{1};Y|X_{2})$ and $\text{Un}(X_{2};Y|X_{1})$ correspond to the *unique* information that is conveyed by $X_{1}$ or $X_{2}$ but not the other, and $\text{Syn}(X_{1},X_{2};Y)$ refers to the *synergistic* information that is provided by $X_{1}$ and $X_{2}$ together but not by each of them separately. For example, consider our two eyes as sources of visual information about the environment. The information that we still have when we close either eye is redundant (e.g. information about colour), while the extra information we derive from combining them (e.g. stereoscopic information about depth) is synergistic. For further reading on PID, we refer the reader to refs. [10,25,26].

### (c) Decomposing information dynamics: From PID to ΦID

As a final piece of mathematical background, we now show how information decomposition can be applied to the temporal evolution of a stochastic dynamical system. Let’s consider two interdependent processes sampled at times t and $t^{\prime }>t$, and denote their corresponding values as $X_{t}^{1},X_{t}^{2}$ and $X_{t^{\prime }}^{1},X_{t^{\prime }}^{2}$, respectively. The information that these two processes carry together from t to $t^{\prime }$ is given by the time-delayed mutual information (TDMI), denoted by $I(X_{t};X_{t^{\prime }})$ where $X_{t}=(X_{t}^{1},X_{t}^{2})$. By regarding $X_{t}^{1}$ and $X_{t}^{2}$ as predictors and the joint future state $X_{t^{\prime }}$ as target, equations (2.1) and (2.2) allow us to decompose the TDMI as follows:

$$\text{TDMI}=\text{Red}(X_{t}^{1},X_{t}^{2};X_{t^{\prime }})+\text{Syn}(X_{t}^{1},X_{t}^{2};X_{t^{\prime }})+\text{Un}(X_{t}^{1};X_{t^{\prime }}|X_{t}^{2})+\text{Un}(X_{t}^{2};X_{t^{\prime }}|X_{t}^{1}).$$

However, this decomposition considers the future state as a single entity and, hence, cannot discriminate between the various ways in which the predictors affect different parts of the target.

**Figure 1.**
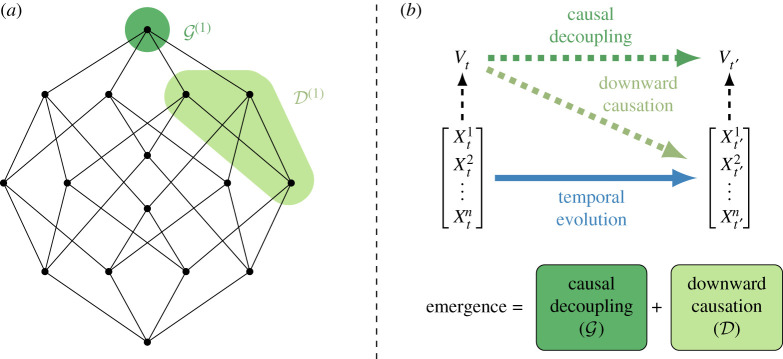
Schematic of the ΦID approach to causal emergence. (*a*) Lattice of ΦID information atoms, with atoms corresponding to causal decoupling (G) and downward causation (D) highlighted. (*b*) Relationship between system variables $X_{t}$, supervenient variables $V_{t}$ and emergent properties (cf. equation (3.2)). Images adapted from [7,27,28]. (Online version in colour.)

This important limitation is overcome by a finer decomposition, called integrated information decomposition (ΦID) [27], which establishes information atoms not only in terms of the relationship between the predictors, but also between the targets (see figure 1). For example, information can be carried redundantly by $X_{t}^{1},X_{t}^{2}$ but received synergistically by $X_{t^{\prime }}^{1},X_{t^{\prime }}^{2}$, which corresponds to a ΦID atom denoted (in simplified notation) by Red→Syn.

By considering these dynamical information atoms, ΦID establishes a way of decomposing PID atoms into a sum of finer ΦID atoms. In particular, each of the four PID atoms can be decomposed into four ΦID atoms, which brings a decomposition of the TDMI into 4×4=16 distinct atoms. For more details about the interpretation of each of the ΦID atoms, and their generalization to more than two time series, we refer the reader to refs. [27,28] (see figure 1).

## 3. Formalizing mereological causal emergence

The first step towards using ΦID to formalize causal emergence is to formalize the notion of *supervenience*. For this purpose, one says that a variable $V_{t}$ is supervenient on the state of the system $X_{t}$ if it is a (possibly noisy) function of $X_{t}$. This definition implies that to have a difference in $V_{t}$ it is necessary for some difference in $X_{t}$ to occur.

Building on this definition, a supervenient feature $V_{t}$ is said to exhibit *causal emergence of order* k if it has predictive power about the future evolution of the underlying system $X_{t}=(X_{t}^{1},\ldots ,X_{t}^{n})$ that is kth-order unique with respect to the state of each part of the system, i.e. if

3.1 $${\text{Un}}^{\left(k\right)}(V_{t};X_{t^{\prime }}|X_{t})>0.$$

The notion of kth-order unique information comes from a PID of n predictors, which generalizes the case of two predictors discussed in the previous section [7, appendix A]. Intuitively, the kth-order unique information ${\text{Un}}^{\left(k\right)}(V_{t};X_{t^{\prime }}|X_{t})$ is the information about $X_{t^{\prime }}$ that $V_{t}$ has access to and no subset of k or fewer parts of $X_{t}$ has access to on its own (although bigger groups may). Causal emergence is, therefore, defined as the capability of some supervenient feature to provide predictive power that cannot be reduced to underlying microscale phenomena—up to order k. Put simply, emergent features have more predictive power than their constituent parts. As an example, consider a bivariate binary system in which the future depends on the parity (i.e. the XOR) of the past [7, fig. 1]. The output of an XOR gate cannot be predicted from either input alone, so a suitably defined feature $V_{t}=X_{t}^{1}⊕X_{t}^{2}$ (where ⊕ denotes the XOR operator) will have greater predictive power than the parts of the system, and thus qualify as an emergent feature.

Crucially, this framework accommodates the coexistence of supervenience and the irreducible predictive power of emergence, which have been previously thought as paradoxical [29,30]. It does so by leveraging the temporal dimension, such that supervenience is operationalized in terms of *instantaneous* relationships (between the system and its observables) and emergence in terms of predictive power *across time*. In this context, a feature could be supervenient without being causally emergent, but not *vice versa*.^1^

One of the main consequences of this theory is that, under relatively general assumptions [7], a system’s capability to display causally emergent features depends directly on how synergistic the system’s dynamics are. Specifically, a system $X_{t}$ possesses causally emergent features of order k if and only if ${\text{Syn}}^{\left(k\right)}(X_{t};X_{t^{\prime }})>0$ [7, theorem 1]. Intuitively, ${\text{Syn}}^{\left(k\right)}(X_{t};X_{t^{\prime }})$ is the information about the future evolution that is provided by the whole system, but is not contained in any set of k or fewer predictors when considered separately from the rest.

This result has two important implications. First, the dependence of emergence on synergistic dynamics suggests one can interpret the term ${\text{Syn}}^{\left(k\right)}(X_{t};X_{t^{\prime }})>0$ as the *emergence capacity* of a system. Second, we can use the formal apparatus of ΦID to decompose ${\text{Syn}}^{\left(k\right)}$ and distinguish two qualitatively different types of emergence:

(i) *Downward causation*, where an emergent feature has unique predictive power over specific parts of the system. Technically, a supervenient feature $V_{t}$ exhibits downward causation of order k over a subsystem of k time series $X^{\alpha }$ if ${\text{Un}}^{\left(k\right)}(V_{t};X_{t^{\prime }}^{\alpha }|X_{t})>0$.
(ii) *Causal decoupling*, in which an emergent feature $V_{t}$ has unique predictive power not over any constituent of size k or less, but on the system as a whole. Technically, a supervenient feature $V_{t}$ exhibits causal decoupling of order k if ${\text{Un}}^{\left(k\right)}(V_{t};V_{t^{\prime }}|X_{t},X_{t^{\prime }})>0$. This corresponds to ‘persistent synergies,’ involving macroscopic variables that have causal influence on other macroscopic variables, above and beyond the microscale effects.

Further derivations show that a system has features that exhibit kth-order downward causation if and only if $D^{\left(k\right)}(X_{t};X_{t^{\prime }})>0$, and has kth-order causally decoupled features if and only if $G^{\left(k\right)}(X_{t};X_{t^{\prime }})>0$, where $D^{\left(k\right)}$ and $G^{\left(k\right)}$ are suitably defined ΦID-based functions (see [7] for details). Moreover, the ΦID framework shows that this taxonomy of emergent phenomena is exhaustive, as the emergence capacity of a system can be decomposed (see figure 1) as

3.2 $${\text{Syn}}^{\left(k\right)}(X_{t};X_{t^{\prime }})=D^{\left(k\right)}(X_{t};X_{t^{\prime }})+G^{\left(k\right)}(X_{t};X_{t^{\prime }}).$$

In summary, these equations imply that causal emergence takes place when groups of variables influence the future of the system together, *but not separately*. Hence, it is not just about counting how many variables predict the system’s future state, but evaluating how they do it.

A final aspect of this theory worth highlighting is that it provides practical measures that are readily computable in large systems. In general, the value of the terms in equations (3.1) and (3.2) depends on a choice of redundancy function,^2^ whose estimation often requires large amounts of data as system size grows. Fortunately, the ΦID formalism of causal emergence enables the derivation of simple measures that provide sufficient criteria for emergence and are independent of the choice of redundancy function. Importantly, these measures are relatively easy to calculate, as they avoid the ‘curse of dimensionality’ since they rely only on kth-order marginals, which are much easier to estimate than the full nth-order joint distribution. This key feature allows the framework to be applicable to a wide range of scenarios, as illustrated by the applications reviewed in §5. More information about these measures can be found in [7].

## 4. Interpretation and remarks

Having considered the main technical elements of the formalism, this section discusses some key aspects of its interpretation while clarifying some potential misunderstandings.

### (a) Interventionist versus probabilistic causation

Some interpretations (e.g. [31]) of the presented framework place emphasis on its relation to the Granger notion of probabilistic causation, as the definition of causal emergence is based on predictive ability—as opposed to, for example, interventionist approaches to causality based on counterfactuals, as proposed by Pearl & Mackenzie [32]. However, it is important to note that the framework presented here belongs to neither the Granger nor Pearl schools of thought, and admits both kinds of causal interpretation depending on the underlying probability distribution from which the relevant quantities are computed. As a matter of fact, all the quantities described in §3 and [7] depend only on the joint probability distribution $p(X_{t^{\prime }},X_{t})$. If this distribution is built using a conditional distribution $p(X_{t^{\prime }}|X_{t})$ that is equivalent to a do() distribution in Pearl’s sense [32], and the system satisfies a few other properties,^3^ then the results of ΦID can be interpreted in an interventionist causal sense. On the other hand, if the distribution is built on purely observational data, then the decomposition obtained from ΦID generally should be understood in the Granger-causal sense (i.e. as referring to predictive ability). In both cases, the formalism developed here applies directly, and it is only the interpretation of the findings that needs to be adapted.

It is also important to clarify that the reason why correlation between variables of a system of interest often does not imply causation is because of hidden (i.e. unobserved) variables. However, if all the relevant variables are measured, then Granger- and Pearl-type analyses coincide. Therefore, we emphasize that while some results might not have an intervention-type interpretation, this is not due to limitations of the formalism in principle but only due to limitations of measurement in practice.

### (b) Lack of invariance under change of coordinates

A possible objection to the framework outlined here is that it critically depends on the specific partition of the underlying system, i.e. on how the *parts* are defined. Put differently, synergy and unique information are not invariant under changes in the way the micro-elements are construed—what is technically known as ‘change of coordinates’.^4^

It is important to remark that this lack of invariance is not a bug, but rather a feature of our framework. Recall that our theory is fundamentally a *mereological* one—i.e. about the relationship between the whole and its parts. Therefore, it is only natural that if the parts change, quantification of the part-whole relationships observed in the system should change too. Put differently, it is reasonable to expect that a mereological account of emergence should critically depend on how the parts are defined, and that any conclusions should be able to change if those parts change.

Following on from §2, we highlight that this property aligns well with the epistemic interpretation of probabilities spearheaded by Jaynes [12]. If one embraces the idea that probabilistic descriptions are representations of states of knowledge, then it follows that the coordinates used to describe the system determine how the joint distribution ought to be marginalized—which is also part of our state of knowledge. Then, it is to be expected that changing the system’s coordinates should change any conclusions drawn from the relationship between marginals—including causal emergence.

### (c) On the order and scale of emergence

Although most of the empirical results from ΦID presented in the literature so far (reviewed in the next section) correspond to emergence of order k=1, it is important to highlight that the formalism allows us to tune the value of k to detect emergence at various spatial scales. In fact, being kth-order emergent implies that there is predictive ability related to interactions of order k+1 or more. In this regard, it is to be noted that a kth-order emergent feature is emergent for all orders j<k, and hence increasing the order makes finding emergent features increasingly challenging. As no system of n parts can display causal emergence of nth order,^5^ an interesting question is to identify the *maximum* k at which emergence takes place—which establishes a characteristic scale for that particular phenomenon.

A related potential misunderstanding is to believe that the ΦID framework for causal emergence only concerns predictive ability at the microscale, without establishing a proper comparison with a macroscale [8]. It is important to clarify that this approach to emergence is established in terms of supervenient macroscopic variables, which may be considered emergent depending on their dynamics and predictive power over the evolution of the system—not too dissimilar from other approaches [5,8]. The fact that dynamical synergy enables the existence of such emergent variables is not an assumption, but a consequence of the theory. Moreover, this result enables a powerful method to characterize emergence: unlike other theories, the ΦID approach to causal emergence can determine the overall capability of a system to host emergent properties without the need to specify any particular macroscopic variable. Further, the ‘scale’ of emergence is tuned by the emergence order k, which sets the measures to focus on high-order interdependencies that do not play a role at scales smaller than k+1 [27].

## 5. Applications

**Figure 2.**
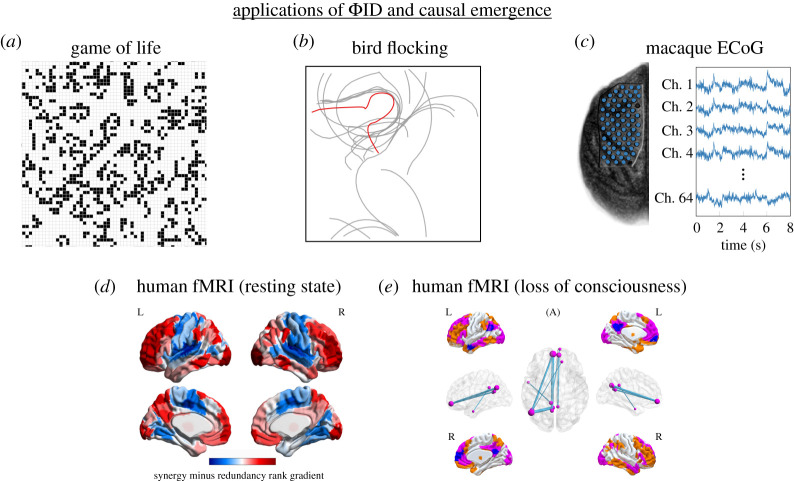
Example published applications of the ΦID approach to causal emergence. Examples include (*a*) Conway’s Game of Life, (*b*) a bird flocking model, (*c*) macaque ECoG during motor control [7], (*d*) human resting-state fMRI brain activity [34] and (*e*) human fMRI during loss of consciousness [35]. Images reproduced from [7,34,35] and the Neurotycho database. (Online version in colour.)

Despite its recent inception, the presented framework has already proven capable of providing insights about a wide range of phenomena (see figure 2). In the following, we first present case studies that demonstrate how the framework aligns with paradigmatic examples of putative emergent behaviour, and then discuss recent results related to the human brain.

This framework provides two approaches to assess emergence in practice: one can (i) test if a given feature of interest has emergent behaviour either directly with the definition (equation 3.1) or via the practical criteria discussed at the end of §3, or one can (ii) calculate the capacity of a system to host *any* emergent feature by computing its dynamical synergy. The latter approach is more encompassing, but requires one to use a redundancy function (see §3) and usually scales poorly with number of parts—making its calculation in large systems very challenging. The former approach focuses on a particular feature, but circumvents those problems allowing one to deal with large systems. In the following, the case studies reviewed in §5a use the practical criteria (i.e. not requiring a choice of redundancy function), while most in §5b calculate dynamical synergy (i.e. requiring a specific redundancy function).

### (a) Confirming intuitions: emergence in the Game of Life and bird flocks

The efficacy of the presented framework to detect emergence was demonstrated in a paradigmatic example of emergent behaviour: Conway’s celebrated Game of Life (GoL) [36]. In GoL, simple local rules determine whether a given cell of a two-dimensional grid will be ON (alive) or OFF (dead) based on the number of ON cells in its immediate neighbourhood. The simple GoL rule nevertheless results in highly complex behaviour, with recognizable self-sustaining structures—known as ‘particles’—that have been shown to be responsible for information transfer and modification [22].

To study emergence in GoL, a ‘particle collider’ was considered in which two particles are set in a colliding course, and the GoL rule is run until the board reaches a steady state [7]. The emergent feature considered, $V_{t}$, was a symbolic, discrete-valued vector that encodes the type of particle(s) present in the board. The ΦID framework (in particular, practical criteria discussed in the previous section) provided a quantitative validation that particles have causally emergent properties, in line with widespread intuition, and further analyses (validated with surrogate data methods) suggested that they may be causally decoupled with respect to their substrate.

Another demonstration of the power of the framework and practical criteria was carried out in a computational model of flocking birds [4,37], another often-cited example of emergent behaviour whereby the flock as a whole arises from the interactions between individuals [7]. Here, the framework showed that the centre of mass can predict its own dynamics better than what can be explained from the behaviour of individual birds (see figure 2).

### (b) Causal emergence in the brain

Moving from simulations to empirical data, the ΦID framework for causal emergence was also adopted to study how motor behaviour might be emergent from brain activity. Simultaneous electrocorticogram (ECoG) and motion capture (MoCap) data of macaques performing a reaching task were analysed, focussing on the portion of neural activity encoded in the ECoG signal that is relevant to predict the macaque’s hand position. Results indicated that the motion-related signal is an emergent feature of the macaque’s brain activity [7].

In the human brain, functional magnetic resonance imaging (fMRI) makes it possible to study non-invasively the patterns of coordinated activity that take place between brain regions. ΦID has been recently adopted to advance the study of brain dynamics, moving beyond simple measures of time series similarity (e.g. Pearson’s correlation or Shannon’s mutual information) to ‘information-resolved’ patterns in terms of ΦID atoms. Remarkably, analyses of human fMRI data have identified a gradient with redundancy-dominated sensory and motor regions at one end, and synergy-dominated association cortices dedicated to multimodal integration and high-order cognition at the other end [34]. Recapitulating the hierarchical organization of the human brain, the synergy-rich regions of the human brain also coincide with regions that have undergone the greatest amounts of evolutionary expansion [34].

In this analysis, the synergistic information is quantified in terms of $G^{\left(k\right)}(X_{t};X_{t^{\prime }})$ (see equation (3.2)) with k=1 calculated over the joint dynamics of pairs of brain areas,^6^ which corresponds to the capacity of those dynamics for causal decoupling (see §3). Therefore, the results reported in [34] indicate that causal emergence (decoupling) increases both along the cortical hierarchy of the human brain, and across the gap from non-human primates to humans.

Relatedly, there has been a long-standing debate on whether consciousness could be viewed as an emergent phenomenon enabled by the complex interactions between neurons. The framework presented here provides ideal tools to rigorously and empirically tackle this question. Moreover, causal decoupling is one of the information atoms of a putative measure of consciousness known as *integrated information* [27], which associates the ability to host consciousness with the extent to which a system’s information is ‘greater than the sum of its parts’ [38]. Interestingly, analysis of fMRI data showed that loss of consciousness due to brain injury corresponds to a reduction of integrated information in the brain [35]. In this way, the more nuanced view on neural information dynamics offered by ΦID holds the promise of further insights for our understanding of consciousness as an emergent phenomenon [28].

## 6. Conclusion

This article presents a review of how recent developments on information decomposition naturally lead to a formal theory of causal emergence. Although this mereological approach to causal emergence is one of many within a rapidly growing field, it has already shown wide applicability across diverse scientific questions. Therefore, the present review sought to bring together the technicalities of the formalism, its interpretation, and results of its practical application, so that each may inform the understanding of the other.

One special feature of this framework is how it allows practical criteria that are applicable to relatively large systems, which opens a broad range of exciting future applications. However, these tools require an explicit feature of interest, whose definition may not be clear in some scenarios of interest (e.g. in resting-state fMRI data). This limitation can be avoided by calculating the capacity of emergence of the dynamics, but the calculation of this scales poorly with the system size—making the calculation of the emergence capacity of large systems (such as highly multivariate brain data) currently unfeasible. Developing procedures to either identify emergent features, or to efficiently calculate emergent capacity in large systems are important avenues for future work.

We hope that the theoretical and empirical advances reviewed in this article may stimulate the growing scientific interest on emergence, which may lead the way towards future breakthroughs on major questions about the role of emergence in the natural world.

## Data Availability

This article has no additional data.

## Footnotes

1 For example, the feature $V_{t}=f(X_{t})=X_{t}^{1}$ is supervenient but not emergent, as it does not predict anything above and beyond individual variables.

2 Multiple redundancy functions exist, and ongoing work is exploring the strengths and weaknesses of different choices. For more information, see [27] and the extensive PID literature.

3 Technically known as faithfulness and causal Markov conditions—see [33] for a detailed description.

4 As a simple example, consider the XOR gate $Y=X^{1}⊕X^{2}$, with $X^{1},X^{2}$ i.i.d. unbiased coin flips, and the change of coordinates $\left(Z^{1},Z^{2})=(X^{1}⊕X^{2},X^{1}\right)$. In this case, $\text{Syn}(X^{1},X^{2};Y)=1$, while $\text{Un}(Z^{1};Y|Z^{2})=1$, showing that information atoms are not invariant under changes of coordinates in general.

5 This mathematical fact implies that this framework does not support phenomena that are not describable by nth-order interactions.

6 The analysis focuses on pairs of areas because currently there is a lack of efficient estimators of $G^{\left(k\right)}(X_{t};X_{t^{\prime }})$ for three or more time series. Developing such estimators is an important avenue for future work.
